# An early predictive model for Kawasaki disease shock syndrome in children in central China

**DOI:** 10.3389/fcvm.2024.1405012

**Published:** 2024-05-27

**Authors:** Yali Wu, Wen Yin, Yini Wen, Jing Chen, Hongxia Tang, Yan Ding

**Affiliations:** Department of Rheumatology and Immunology, Wuhan Children’s Hospital (Wuhan Maternal and Child Healthcare Hospital), Tongji Medical College, Huazhong University of Science & Technology, Wuhan, China

**Keywords:** Kawasaki disease, Kawasaki disease shock syndrome, predictive scoring model, NLR, fibrinogen, serum sodium

## Abstract

**Introduction:**

This study aims to analyze the clinical features of Kawasaki disease (KD) shock syndrome (KDSS) and explore its early predictors.

**Methods:**

A retrospective case–control study was used to analyze KD cases from February 2016 to October 2023 in our hospital. A total of 28 children with KDSS and 307 children who did not develop KDSS were included according to matching factors. Baseline information, clinical manifestations, and laboratory indicators were compared between the two groups. Indicators of differences were analyzed based on univariate analysis; binary logistic regression analysis was used to identify the risk factors for KDSS, and then receiver operating characteristic analysis was performed to establish a predictive score model for KDSS.

**Results:**

Elevated neutrophil-to-lymphocyte ratio(NLR) and decreased fibrinogen (FIB) and Na were independent risk factors for KDSS; the scoring of the above risk factors according to the odds ratio value eventually led to the establishment of a new scoring system: NLR ≥ 7.99 (6 points), FIB ≤ 5.415 g/L (1 point), Na ≤ 133.05 mmol/L (3 points), and a total score of ≥3.5 points were high-risk factors for progression to KDSS; otherwise, they were considered to be low-risk factors.

**Conclusion:**

Children with KD with NLR ≥ 7.99, FIB ≤ 5.415 g/L, and Na ≤ 133.05 mmol/L, and those with two or more of the above risk factors, are more likely to progress to KDSS, which helps in early clinical diagnosis and treatment.

## 1 Introduction

Kawasaki disease (KD) is an acute vasculitis of unknown etiology involving small- and medium-sized arteries first reported by Dr. Kawasaki in 1967 (1). It has become the leading cause of acquired heart disease in children in developed countries (2, 3). The main manifestations of children are persistent fever, conjunctival congestion, acute non-cervical lymphadenopathy, lip redness, rhagadia, and edema of the extremities (4). The most significant complication is coronary artery lesions (CALs), which can lead to a poor prognosis of coronary artery dilatation, coronary aneurysm, myocardial infarction, and sudden death (2, 5). The standard treatment for this disease typically involves a single intravenous injection of immunoglobulin (IVIG) at a dose of 2 g/kg combined with oral aspirin, which has been shown to reduce the risk of coronary artery damage from 25% to 5% (2).

In 1975, Kato et al. (6) reported a case of shock, heart failure, and other serious complications in a 6-month-old child with KD. Since then, this critical KD with hemodynamic changes has been reported by successive investigators (7). In 2009, Kanegaye et al. (8) defined, for the first time, the Kawasaki disease shock syndrome (KDSS) as a condition that manifests during the acute phase of KD in children, characterized by circulatory and hemodynamic disorders, hypotension, or the need for vasoactive drug infusion. Untimely treatment can be life-threatening, but its predisposing factors and pathogenesis remain unknown. It is widely recognized that during the acute phase of KD, there is an increased vascular permeability and excessive release of cellular inflammatory factors, which is complicated by severe capillary leakage, reduced peripheral vascular resistance, and myocardial contractile dysfunction. Other intersecting factors and pathophysiological processes impact each other, which accelerates the occurrence of circulatory disorders (9). The prevalence of KDSS in children with KD is approximately 1.23%–7.00% (8, 10, 11), and the probability of coronary artery dilatation and the development of giant coronary artery aneurysms is significantly higher compared with that of ordinary KD (9, 12), which is more prone to cardiac involvement, such as acute myocarditis, left ventricular insufficiency, pericardial effusion, electrocardiographic abnormalities, and, in severe cases, transient heart failure (12, 13). There is evidence showing that the prognosis of KDSS is correlated with the degree of coronary damage and the severity of complications (13, 14); hence, early diagnosis and treatment are crucial. However, the low incidence of KDSS, atypical early clinical manifestations, and lack of unified diagnostic criteria increase the risk of missed and misdiagnosed cases. Therefore, it is necessary to explore the predictive model of this disease for early diagnosis and treatment to improve the prognosis.

By analyzing the clinical characteristics of children with KDSS and those who did not progress to KDSS, this study sought to explore the early predictors of KDSS in children.

## 2 Materials and methods

### 2.1 Study population

This study was a retrospective case–control study approved by the Institutional Review Board of Wuhan Children's Hospital, Tongji Medical College, Huazhong University of Science and Technology (No. 52022R053-E01). Children diagnosed with KD in the Department of Rheumatology and Immunology of our hospital from February 2016 to October 2023 were included.

The KD inclusion criteria were as follows: (1) those who met the diagnostic criteria for KD published by the American Heart Association (AHA) in 2017 and (2) those with classic KD diagnosed in the presence of fever for at least 5 days (the day of fever onset is considered the first day of fever), together with at least four of five principal clinical features: (1) erythema and cracking of lips, strawberry tongue, and/or erythema of oral and pharyngeal mucosa; (2) bilateral bulbar conjunctival injection without exudate; (3) rash, maculopapular, diffuse erythroderma, or erythema multiforme-like; (4) erythema and edema of the hands and feet in acute phase and/or periungual desquamation in subacute phase; and (5) cervical lymphadenopathy (≥1.5 cm diameter, usually unilateral). In cases where ≥4 principal clinical features are present, particularly when redness and swelling of the hands and feet are observed, KD diagnosis can be made with 4 days of fever, complete data, age <18 years, and those who were admitted to the hospital within 10 days of onset of fever and treated with a single dose of IVIG (2 g/kg) and oral aspirin (30–50 mg/kg/d).

The KDSS inclusion criteria were as follows: those with KDSS diagnosed in accordance with the Kanegaye criteria of 2009 (8) if the sustained presence of any of the following conditions caused the treating clinicians to initiate volume expansion, vasoactive agent infusion, or transfer to an intensive care setting: systolic hypotension for age (infants aged 0–28 days, <60 mmHg; infants aged 1–12 months, <70 mmHg; children aged 1–10 years, <70 + [2 × age] mmHg; youths aged >10 years, ≤90 mmHg); a decrease in the systolic blood pressure from baseline of ≥20%; or clinical signs of poor perfusion (such as tachycardia, prolonged capillary filling time, cool extremities, diminished pulses, oliguria, or mental status changes not accounted for by other conditions, such as fever or ambient temperature), regardless of the measured blood pressure.

The KD exclusion criteria included those with (1) incomplete clinical data; (2) sepsis with positive blood cultures; (3) comorbid cardiovascular underlying diseases, such as congenital heart disease, heart failure, and essential hypertension; (4) loss of visitors; and (5) immunodeficiency.

**Figure 1:**
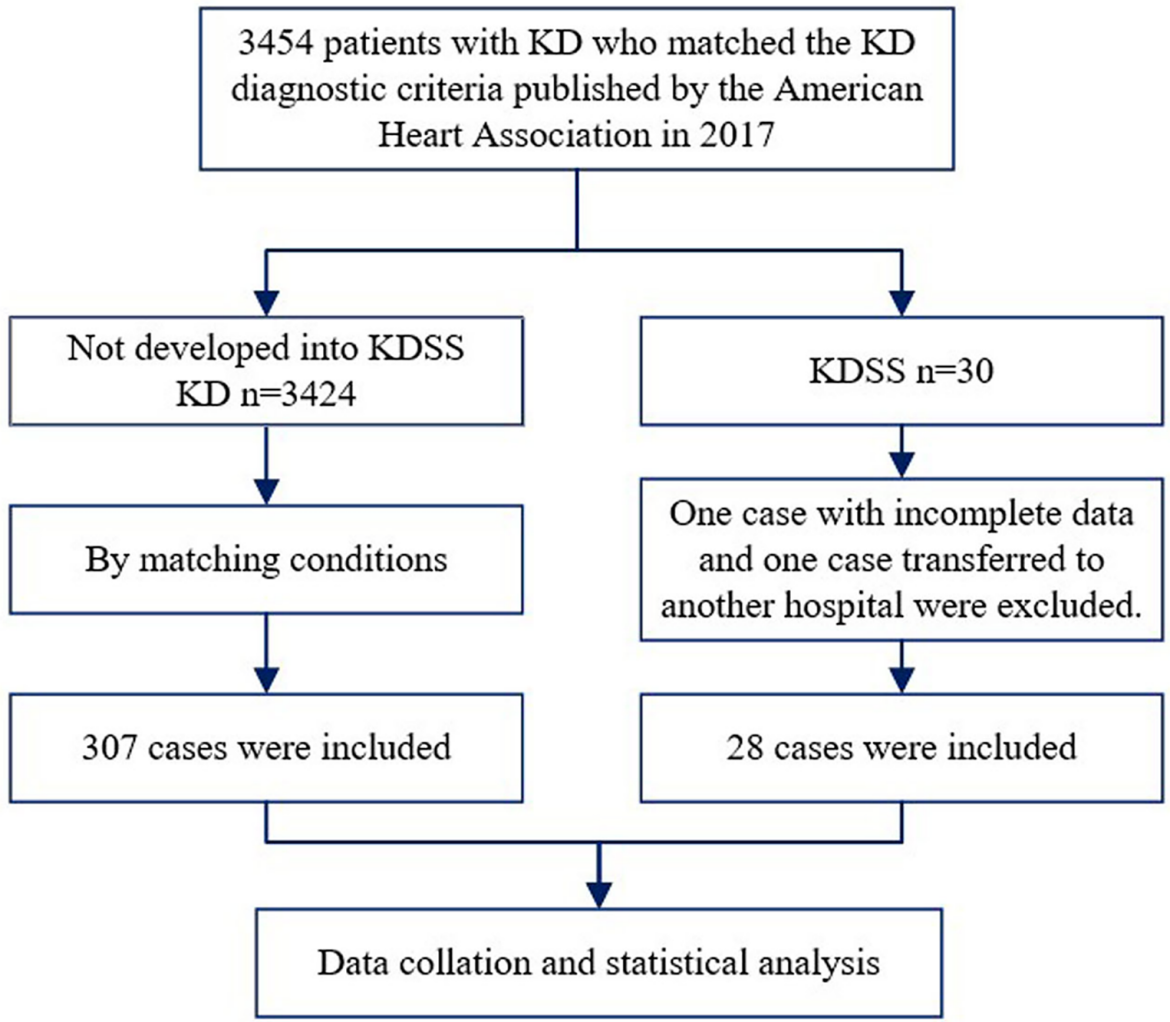
Flowchart of the patient selection process. KD, Kawasaki disease; KDSS, Kawasaki disease shock syndrome.

The included children were categorized into KD and KDSS groups based on the clinical outcomes. Twenty-eight children who met the criteria for KDSS and 11 children who did not develop KDSS were selected as control cases for each patient, using time of admission (±1 week) as a matching factor and excluding the effect of seasonal factors, resulting in 307 children with KD (15) (Figure 1).

### 2.2 Data collection

The medical record system and data platform of Wuhan Children's Hospital acquired the information of 335 patients prior to IVIG treatment. The data collected included the following: (1) general demographics, such as gender, age, and body mass index (BMI); (2) clinical manifestations, such as fever duration, rash, conjunctivitis, lip and tongue changes, lymphadenopathy, and extremity changes; (3) laboratory results, such as white blood cells (WBC), hemoglobin (Hb), platelets (PLT), neutrophil-to-lymphocyte ratio [NLR = neutrophils (×109/L)/lymphocytes (×109/L)], C-reactive protein (CRP), procalcitonin (PCT), erythrocyte sedimentation rate (ESR), serum ferritin (SF), fibrinogen (Fib), aspartate aminotransferase-to-alanine aminotransferase ratio (AST/ALT), lactate dehydrogenase (LDH), creatine kinase-MB (CK-MB), serum albumin (ALB), globulin (GLO), serum immunoglobulin (Ig), total bilirubin (TBIL), serum creatinine (Scr), blood urea nitrogen (BUN), activated partial thromboplastin time (APTT), prothrombin (PT), serum sodium (Na), serum kalium (K), CD3^+^CD4^+^ T, CD3^+^CD8^+^ T, CD19^+^ B, IgE, IgM, IgG, IgA, IL-2, IL-4, IL-6, IL-10, IFN-γ, and TNF-α; and (4) cardiac ultrasound, such as coronary artery *Z*-value, valve regurgitation, ventricular enlargement, decreased cardiac function, and hydropericardium. The data were entered into Excel tables and checked by two trained physicians.

### 2.3 Statistical analysis

Data analysis was performed using SPSS Statistics version 22 and R version 4.1.3. The measurement data were tested for normality, and the non-normally distributed measurement data were expressed as M (Q1, Q3), with comparisons between groups made by the Mann–Whitney *U*-test. The count data were expressed as the number of cases and compared between groups using the four-compartment table *χ*^2^ test. All differences were considered statistically significant at *p* < 0.05. The differential variables were subjected to a one-way logistic analysis, and *p* < 0.05 was included in a multifactorial logistic model to screen the valuable risk factors and score them according to OR values to establish a predictive scoring model. The total score was calculated for each group of patients, and the maximum Youden index corresponding to the total score cutoff value, sensitivity, and specificity was derived using the subject receiver operating characteristic (ROC) curve. Statistical significance was set at *p* < 0.05. The low prevalence of KDSS and the small number of included cases required the use of leave-one-out cross-validation to assess the performance of logistic regression models. The models with an area under curve (AUC) of >0.7 were considered clinically valuable.

## 3 Results

### 3.1 General demographic data and clinical presentation in the KD and KDSS groups

**Figure 2:**
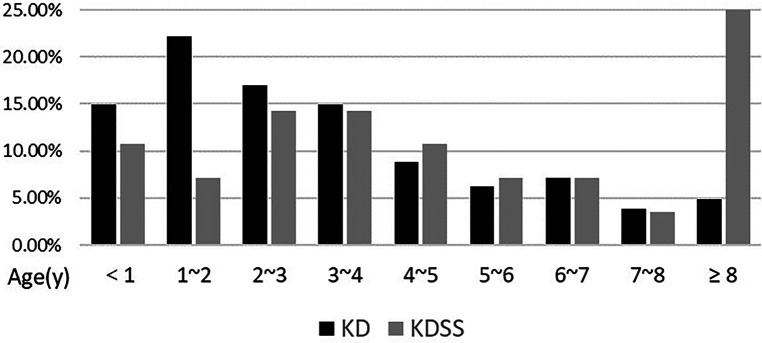
Age distribution between the two groups.

**Figure 3:**
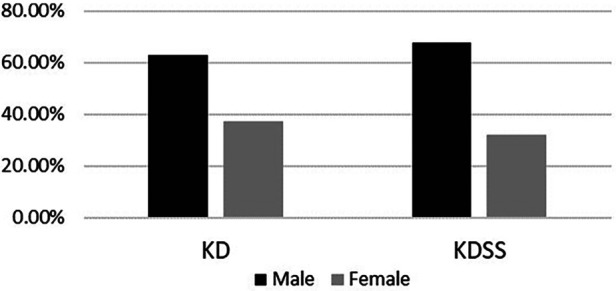
Gender distribution between the two groups.

**Figure 4:**
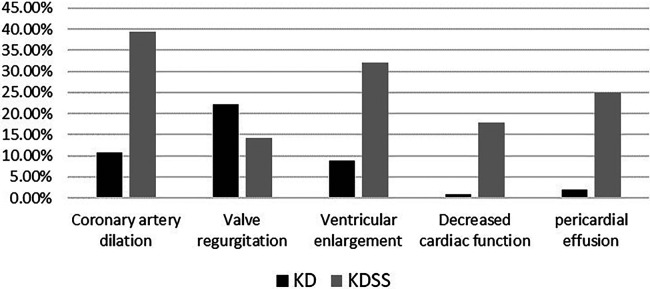
Distribution of cardiac ultrasound performance between the two groups.

Among the 3,454 children with KD included, 30 were diagnosed with KDSS (*n* = 28, 0.87%). One case with incomplete information and one referral were excluded, resulting in a total of 28 KDSS enrolled in this study. A total of 307 children with KD were included as a control group according to matching factors. Male children predominated in both groups (KDSS *n* = 19, 67.9%, vs. KD *n* = 193, 62.9%; *p *= 0.6), and children in the KDSS group were older than those in the KD group [KDSS 4.53 (2.38, 8.15) vs. KD 2.69 (1.46, 4.82), *p *< 0.05], had a higher BMI [KDSS 17.3 (15.42, 18.35) vs. KD 15.63 (14.4, 16.87), *p *< 0.05] and a longer duration of fever [KDSS 6 (5, 7.75) vs. KD 5 (5, 6), *p *< 0.05], and were more likely to have CALs (KDSS *n* = 11, 39.29% vs. KD *n* = 33, 10.75%; *p *< 0.05), ventricular enlargement (KDSS *n* = 9, 32.14% vs. KD *n* = 27, 8.79%; *p *< 0.05), decreased cardiac function (KDSS *n* = 5, 17.86% vs. KD *n* = 3, 0.98%; *p* < 0.05), and pericardial effusion (KDSS *n* = 7, 25% vs. KD *n* = 6, 1.95%; *p *< 0.05). There were no statistically significant differences observed in rash, conjunctival congestion, lip and tongue changes, neck lymph node enlargement, terminal changes in the limbs, and alvular regurgitation between the two groups (Table 1 and Figures 2–4).

**Table 1:** Baseline, clinical presentation, and cardiac ultrasound performance in the KD and KDSS groups.

Items	KD	KDSS	*U*/*χ*^2^	*p*
No. of patients	307	28		* *
Gender (male)	193	19	0.275	0.6
Age (years)	2.69 (1.46, 4.82)	4.53 (2.38, 8.15)	−2.812	0.0049251
BMI	15.63 (14.4, 16.87)	17.3 (15.42, 18.35)	−3.113	0.0018547
Duration of fever (days)	5 (5, 6)	6 (5, 7.75)	−2.505	0.012
Rash (*n*)	245	24	0.566	0.452
Conjunctivitis (*n*)	273	24	0.263	0.608
Lip and tongue changes (*n*)	260	26	1.37	0.242
Lymphadenopathy (*n*)	210	16	1.482	0.223
Extremity changes (*n*)	219	19	0.151	0.698
Coronary artery dilation (*n*)	33	11	18.315	0.000
Valve regurgitation (*n*)	68	4	0.94	0.332
Ventricular enlargement (*n*)	27	9	15.584	0.000
Decreased cardiac function (*n*)	3	5	24.542	0.000
Pericardial effusion (*n*)	6	7	30.619	0.000

KD, Kawasaki disease; KDSS, Kawasaki disease shock syndrome; BMI, body mass index.

### 3.2 Laboratory data of patients with KDSS or KD

The comparison of 38 laboratory data between the KD and KDSS groups showed that children in the KDSS group had significantly higher NLR, CRP, PCT, Ferr, TBIL, Cr, BUN, APTT, PT, IgE, IgG, IL-6, and IL-10 (*p *< 0.05), and HGB, PLT, Fib, CK-MB, ALB, Na, K, CD3^+^CD4^+^ T, CD3^+^CD8^+^ T, CD19^+^ B, IL-4, and TNF-α were significantly decreased (*p* < 0.05) (Tables 2, 3).

**Table 2:** Comparison of the blood routine, inflammatory indices, biochemical results, and coagulation function between the KD and KDSS groups.

Variables	KD	KDSS	*U*/*t*	*p*
WBC (10^9^/L)	11.52 (8.57, 14.86)	16.45 (5.79, 24.71)	−1.277	.202
Hb (g/L)	108 (100, 114)	99.5 (95, 110.75)	−2.626	.009
PLT count (10^9^/L)	353 (273, 446)	187.5 (87.25, 284.5)	−5.415	.000
NLR	2.7 (1.43, 5)	11.24 (4.06, 18.41)	−5.291	.000
CRP (mg/L)	52.1 (34.5, 96.2)	112.5 (60.68, 134.5)	−3.546	.000
PCT (ng/ml)	0.47 (0.21, 1.51)	4.02 (1.46, 11.79)	−6.186	.000
ESR (mm/h)	47.23 (23, 69)	48 (29.5, 68.25)	−.279	.780
SF (ng/ml)	150.89 (104.52, 209.84)	340.82 (171.83, 917.01)	−5.032	.000
Fib (g/L)	5.53 ± 1.55	4.90 ± 1.84	2.019	.044
ALT (U/L)	20 (11, 50)	22.5 (14.5, 73.25)	−.760	.447
AST (U/L)	30 (22, 48)	29 (20.25, 56.25)	−.205	.838
AST/ALT	1.57 (0.79, 2.29)	1.27 (0.68, 1.98)	−1.063	.288
LDH (U/L)	304 (253, 387)	285 (212.5, 441)	−.900	.368
CK-MB (U/L)	28 (20, 41)	18.5 (13.25, 34.75)	−3.013	.003
ALB (g/L)	38.6 (35.5, 41.2)	32 (26.75, 35.7)	−5.132	.000
GlO (g/L)	22.6 (20, 26.3)	22 (20.13, 25.73)	−.352	.725
TBIL (µmol/L)	5.7 (4.2, 8.9)	8.7 (6.35, 18.2)	−3.052	.002
Scr (µmol/L)	25.5 (21.2, 30.9)	45.05 (32.2, 59.55)	−6.156	.000
BUN (µmol/L)	3.1 (2.4, 3.8)	4.65 (2.98, 6.1)	−4.370	.000
APTT (s)	32.9 (29.2, 37.8)	39.25 (31.68, 43.88)	−3.551	.000
PT (s)	12.1 (11.4, 13)	12.5 (12.03, 13.55)	−2.197	.028
Na (mmol/L)	137.45 ± 2.96	132.79 ± 3.94	7.733	.000
K (mmol/L)	4.25 (3.83, 4.75)	3.56 (3.14, 4)	−4.618	.000

KD, Kawasaki disease; KDSS, Kawasaki disease shock syndrome; WBC, white blood cells; Hb, hemoglobin; PLT, platelets; NLR, neutrophil-to-lymphocyte ratio; CRP, C-reactive protein; PCT, procalcitonin; ESR, erythrocyte sedimentation rate; SF, serum ferritin; Fib, fibrinogen; AST/ALT, aspartate aminotransferase-to-alanine aminotransferase ratio; LDH, lactate dehydrogenase; CK-MB, creatine kinase-MB; ALB, serum albumin; GLO, globulin; TBIL, total bilirubin; Scr, serum creatinine; BUN, blood urea nitrogen; APTT, activated partial thromboplastin time; PT, prothrombin; Na, serum sodium; K, serum kalium.

**Table 3:** Comparison of the cellular immunity, humoral immunity, and cytokine results between the KD and KDSS groups.

Variables	KD	KDSS	*U*/*t*	*p*
CD3^+^CD4^+^ T cells (cells/µl)	341 (125, 899)	218.5 (64.25, 324.25)	−2.472	.013
CD3^+^CD8^+^ T cells (cells/µl)	549 (303, 766)	150 (114.5, 269)	−5.405	0.000
CD19^+^ B cells (cells/µl)	804 (486, 1,213)	334.5 (152, 727)	−4.193	.000
IgE (g/L)	84.5 (28, 166.84)	166.84 (90.1, 316)	−3.156	.002
IgM (g/L)	0.97 (0.71, 1.3)	0.9 (0.73, 1.08)	−.514	.607
IgG (g/L)	7.07 (5.47, 8.59)	8.14 (6.24, 12.33)	−2.116	.034
IgA (g/L)	0.73 (0.43, 1.16)	0.97 (0.51, 1.35)	−1.142	.254
IL2 (pg/ml)	4.14 (2.93, 5.76)	3.39 (1.85, 5.41)	−1.285	.199
IL4 (pg/ml)	4.11 (3.3, 5)	3.09 (1.82, 4.4)	−3.376	.001
IL6 (pg/ml)	123.95 (43.76, 237.9)	233.9 (53.17, 636.14)	−2.147	.032
IL10 (pg/ml)	12.21 (6.51, 24.71)	34.92 (15.32, 68.11)	−3.953	.000
IFN-γ (pg/ml)	6.82 (3.97, 15.78)	6.07 (3.34, 18.34)	−.399	.690
TNF-α (pg/ml)	5.14 (2.98, 7.15)	3.51 (2.16, 5.8)	−2.296	.022

Ig, serum immunoglobulin; IL, interleukin; IFN, interferon; TNF, tumor necrosis factor.

### 3.3 Early predictors of progression to KDSS in children

A one-way logistic regression analysis of each of the abovementioned variables with differences found that older age; longer duration of fever; higher NLR, NLR, CRP, PCT, Ferr, Cr, BUN, APTT, IgG, IL-6, and IL-10; and decreased HGB, PLT, Fib, CK-MB, ALB, Na, K, CD3^+^CD8^+^ T, CD19^+^ B, IL-4, and TNF-α were predictors of KDSS. A multifactorial logistic regression analysis of 24 early predictors found that elevated NLR and decreased Fib and Na were independent predictors of KDSS (Table 4).

**Table 4:** Logistic regression analysis to identify independent risk factors for predicting KDSS.

Variables	Univariate		Multivariate	
	Crude OR (95% CI)	*p*	Adjusted OR (95% CI)	*p*
Gender	1.24 (1.101, 1.396)	.000		
BMI	1.088 (0.98, 1.209)	.115		
Duration of fever (days)	1.348 (1.084, 1.675)	.007		
Hb	0.947 (0.912, 0.983)	.005		
PLT	0.989 (0.986, 0.993)	.000		
NLR	1.22 (1.137, 1.309)	.000	1.333 (1.036, 1.716)	.026
CRP	1.012 (1.005, 1.019)	.001		
PCT	1.294 (1.166, 1.436)	.000		
SF	1.006 (1.004, 1.008)	.000		
Fib	0.774 (0.602, 0.996)	.046	0.202 (0.055, 0.733)	.015
CK-MB	0.951 (0.916, 0.986)	.007		
ALB	0.776 (0.71, 0.848)	.000		
TBIL	1.018 (1, 1.035)	.050		
Scr	1.15 (1.099, 1.202)	.000		
BUN	2.019 (1.529, 2.666)	.000		
APTT	1.106 (1.054, 1.16)	.000		
PT	1.282 (0.97, 1.695)	.081		
Na	0.662 (0.579, 0.758)	.000	0.592 (0.366, 0.956)	.032
K	0.217 (0.11, 0.427)	.000		
CD3^+^CD4^+^ T	0.999 (0.998, 1)	.052		
CD3^+^CD8^+^ T	0.995 (0.993, 0.997)	.000		
CD19^+^ B	0.998 (0.997, 0.999)	.003		
IgE	1.001 (1, 1.002)	.117		
IgG	1.109 (1.026, 1.198)	.009		
IL4	0.614 (0.464, 0.812)	.001		
IL6	1.001 (1, 1.001)	.014		
IL10	1.011 (1.004, 1.018)	.002		
TNF-α	0.854 (0.735, 0.992)	.039		

KDSS, Kawasaki disease shock syndrome; BMI, body mass index; Hb, hemoglobin; PLT, platelets; NLR, neutrophil-to-lymphocyte ratio; CRP, C-reactive protein; PCT, procalcitonin; SF, serum ferritin; Fib, fibrinogen; CK-MB, creatine kinase-MB; ALB, serum albumin; TBIL, total bilirubin; Scr, serum creatinine; BUN, blood urea nitrogen; APTT, activated partial thromboplastin time; PT, prothrombin; Na, serum sodium; K, serum kalium.

### 3.4 Scoring modeling

**Figure 5:**
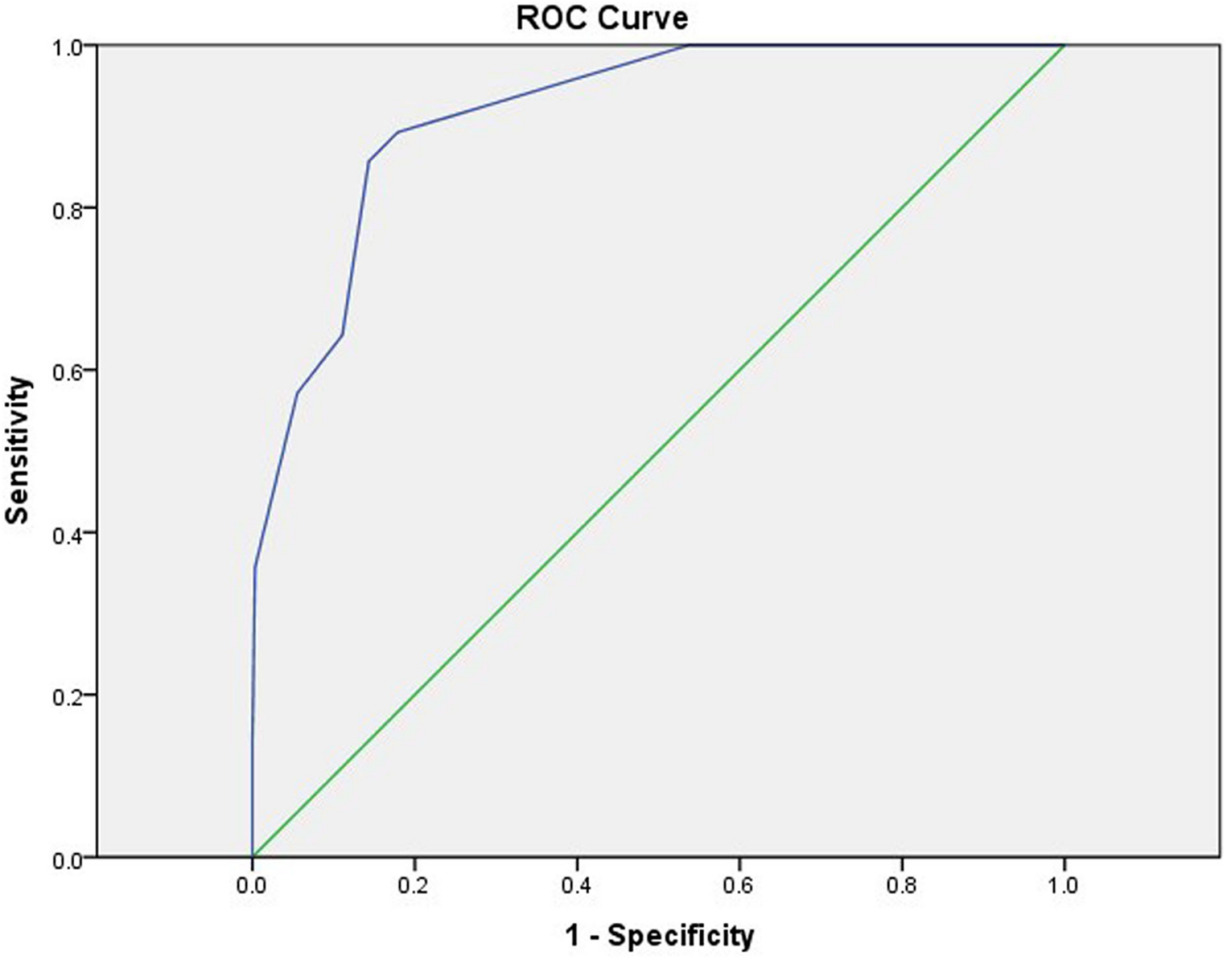
ROC curves for predicting KDSS using the new scoring model. AUC = 0.916 (95% CI, 0.870–0.962). ROC, receiver operating characteristic; KDSS, Kawasaki disease shock syndrome; AUC, area under ROC curve; CI, confidence interval.

The cutoff values of NLR, Fib, and Na were obtained by the ROC curve analysis, and a score was assigned to each variable based on the OR: NLR ≥ 7.99 (6 points), Fib ≤ 5.415 g/L (1 point), and Na ≤ 133.05 mmol/L (3 points). The sensitivities were 0.643, 0.679, and 0.607, and the specificities were 0.889, 0.557, and 0.928. The total score of each enrolled KD child was calculated and analyzed using ROC curve analysis, which revealed that a total score ≥3.5 points indicated a high risk for progression to KDSS (AUC 0.916), whereas a lower score indicated a low risk. The AUC for applying the leave-one-out cross-validation was 0.872; sensitivity, 0.958; and specificity, 0.607 (Table 5 and Figure 5).

**Table 5:** Cutoff values and points after continuous variables converted to dichotomous variables.

Variables	Cutoff value	Sensitivity	Specificity	OR (95% CI)	*p*	Point
NLR	7.990	.643	0.889	1.333 (1.036, 1.716)	.026	6 (≥7.99), 0 (<7.99)
Fib	5.415	.679	0.557	0.202 (0.055, 0.733)	.015	1 (≤5.415), 0 (>5.415)
Na	133.050	.607	0.928	0.592 (0.366, 0.956)	.032	3 (≤133.05), 0 (>133.05)

NLR, neutrophil-to-lymphocyte ratio; Fib, fibrinogen; Na, serum sodium; OR, odds ratio.

## 4 Discussion

KDSS occurs most often in the acute phase of KD (16, 17) and is prone to multiorgan impairment, especially in the blood-rich heart and its vessels (2), due to short-term cytokine dysregulation, myocardial dysfunction, and vasculitis with persistent capillary leakage (12, 13). In 2018, Gamez-Gonzalez et al. (18) reported 12 cases of KDSS from two Mexican hospitals and retrieved 91 cases of KDSS between 1993 and 2016 for a meta-analysis stating that 72.8% of children had coronary artery abnormalities (CAA), 65% of which had coronary aneurysms, and 4.8% developed giant aneurysms. Accordingly, 27.2% of children had valvular regurgitation most frequently in the mitral valve (13.6%) and pericardial effusion in 20.4%. Ejection fraction <50% was found in 44.6% of the children, and 49.5% had a combined heart failure. A study by Li et al. (19) found that coronary aneurysms occurred in 55.56% of children with KDSS. A higher percentage developed acute myocarditis, left ventricular insufficiency, valvular regurgitation, pericardial effusion, ECG abnormalities, and, in severe cases, transient heart failure (12, 13). Our study similarly confirmed that children with KDSS had a higher risk of coronary dilatation (39.29%), ventricular enlargement (32.14%), decreased cardiac function (17.86%), and pericardial effusion (25%). However, valvular regurgitation was found in 14.29% of children, and the difference was not statistically significant compared to children who did not progress to KDSS.

We explored the risk factors of KDSS while establishing the first KDSS score prediction model in central China, that is, NLR ≥ 7.99 (6 points), Fib ≤ 5.415 g/L (1 point), and Na ≤ 133.05 mmol/L (3 points). A total score of ≥3.5 points indicated a high risk for progression to KDSS, whereas a lower score indicated a low risk. The model had an AUC of 0.916 and good predictive efficacy (Figure 5). The highest score NLR in this predictive scoring model was 6. The reason may be that neutrophils play a key role in the elicitation of pro-inflammatory responses *in vivo* (20–22), that lymphocytes play a moderating role in these states, that lymphocyte counts are usually reduced in pro-inflammatory states (23), and that, consequently, the inverse relationship between neutrophils and lymphocytes in pro-inflammatory states results in a significantly higher NLR. Chidambaram et al. (24) concluded that NLR is an independent predictor of CAL in patients with KD, and an NLR ≥ 2.08 on days 4–6 post-febrile identified children at risk for CALs. In a meta-analysis conducted by Sarejloo et al. (25), which analyzed 6,334 cases of KD from 17 articles, of which 1,328 developed CAA, it was found that patients with coronary artery aneurysms had significantly higher levels of NLR than those of patients without coronary artery aneurysms and that NLR may be useful in monitoring the development of CAA in these patients and may further imply that it mediates the pathogenesis of this CAA in underlying inflammation. In our study, NLR ≥ 7.99 was a predictor of the occurrence of KDSS with a sensitivity of 0.674 and a specificity of 0.889. To the best of our knowledge, this is the first time that NLR has been shown to be an early predictor in KDSS.

This predictive scoring model has Fib ≤ 5.415 g/L as 1. It is well known that decreased Fib is one of the diagnostic criteria for systemic juvenile idiopathic arthritis combined with macrophage activation syndrome in children (26) and a diagnostic criterion or supportive basis for hemophagocytic lymphohistiocytosis in 2004 (27) and 2009 (28). This may be related to the decreased fibrinogen synthesis by the liver in a hyperinflammatory state. It has also been reported that decreased fibrinogen is involved in severe complications, such as disseminated intravascular coagulation (DIC) and thrombotic microangiopathy (TMA) (29). A reduced Fib was confirmed as a predictor of the combined macrophage activation syndrome in the study of KD by Wen et al. (30). Our current study found that decreased fibrinogen was a risk factor for KDSS despite the small score; hence, we hypothesize that Fib may be involved in the hyperinflammatory response to KD and play a role in the progression to KDSS.

Our study showed that hyponatremia was more common in KDSS, which has been reported in previous studies. A case–control study by Schuster et al. (31) at the Ann & Robert H. Lurie Children's Hospital of Chicago found that hyponatremia is a feature of KDSS. In a meta-analysis that included 13 studies, Zheng et al. found significant differences in hyponatremia and factors, such as white blood cell counts, platelet counts, and ALT, in children in the KDSS group compared to the KD group (17). The pathophysiologic mechanisms of hyponatremia in patients with KDSS can include dehydration, inappropriate antidiuretic hormone secretion syndrome, and acute kidney injury (31, 32). Hyponatremia can contribute to the presence of shock because of increased vascular leakage or poor cardiac function. In this study, hyponatremia less than or equal to 133.05 mmol/L was found to be an independent risk factor for predicting the development of KDSS, and this threshold needs to be taken into account by clinicians.

However, this predictive scoring model has some limitations. First, this is a retrospective study, in which all test data were sampled within 24 h after the children were admitted to the hospital, at which time, the duration of fever was inconsistent for each KD child, which may result in biased results. Furthermore, this is a single-center case–control study with a small sample size of children with KDSS enrolled, which may lead to bias in the results due to the differences in geography and race; therefore, a subsequent inclusion of a multicenter and large sample is needed to validate the model.

## 5 Conclusion

In this study, we confirmed that KDSS is more likely to lead to cardiac and coronary complications. Additionally, we established a new predictive scoring system for KDSS in central China, that is, NLR ≥ 7.99 (6 points), Fib ≤ 5.415 g/L (1 point), and Na ≤ 133.05 mmol/L (3 points), with a total score of ≥3.5 points for high risk and a lower score for low risk, which is a good prediction of the effectiveness of the scoring model. This is expected to guide the clinical treatment of KDSS and reduce the occurrence of poor prognosis.

## Data Availability

The original contributions presented in the study are included in the article/Supplementary Material; further inquiries can be directed to the corresponding authors.
